# An *H_∞_* Strategy for Strain Estimation in Ultrasound Elastography Using Biomechanical Modeling Constraint

**DOI:** 10.1371/journal.pone.0073093

**Published:** 2013-09-13

**Authors:** Zhenghui Hu, Heye Zhang, Jinwei Yuan, Minhua Lu, Siping Chen, Huafeng Liu

**Affiliations:** 1 State Key Laboratory of Modern Optical Instrumentation, Department of Optical Engineering, Zhejiang University, Hangzhou, Zhejiang, China; 2 Key Lab for Health Informatics of the Chinese Academy of Sciences, Shenzhen Advanced Institutes of Technology, Chinese Academic of Sciences, Shenzhen, China; 3 National-Regional Key Technology Engineering Laboratory for Medical Ultrasound, Guangdong Key Laboratory for Biomedical Measurements and Ultrasound Imaging, Department of Biomedical Engineering, School of Medicine, Shenzhen University, Shenzhen, China; Glasgow University, United Kingdom

## Abstract

The purpose of ultrasound elastography is to identify lesions by reconstructing the hardness characteristics of tissue reconstructed from ultrasound data. Conventional quasi-static ultrasound elastography is easily applied to obtain axial strain components along the compression direction, with the results inverted to represent the distribution of tissue hardness under the assumption of constant internal stresses. However, previous works of quasi-static ultrasound elastography have found it difficult to obtain the lateral and shear strain components, due to the poor lateral resolution of conventional ultrasound probes. The physical nature of the strain field is a continuous vector field, which should be fully described by the axial, lateral, and shear strain components, and the clinical value of lateral and shear strain components of deformed tissue is gradually being recognized by both engineers and clinicians. Therefore, a biomechanical-model-constrained filtering framework is proposed here for recovering a full displacement field at a high spatial resolution from the noisy ultrasound data. In our implementation, after the biomechanical model constraint is integrated into the state-space equation, both the axial and lateral displacement components can be recovered at a high spatial resolution from the noisy displacement measurements using a robust 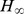 filter, which only requires knowledge of the worst-case noise levels in the measurements. All of the strain components can then be calculated by applying a gradient operator to the recovered displacement field. Numerical experiments on synthetic data demonstrated the robustness and effectiveness of our approach, and experiments on phantom data and *in-vivo* clinical data also produced satisfying results.

## Introduction

The routine clinical practice of palpation represents a qualitative assessment of tissue stiffness based on the significant difference in elastic properties between normal and diseased tissues [1], [2]. However, manual palpation is considered to be subjective, inaccurate, and highly operator-dependent, especially in detecting small and/or deeply located pathological lesions. During the past 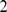 decades, a number of elasticity imaging techniques have been developed for measuring the elasticity of tissues quantitatively, using ultrasound [3]–[10], magnetic resonance imaging [11]–[13], and other imaging modalities [14], [15]. Compared with conventional morphological images, images of elasticity are able to display the distribution of stiffness/elastic properties of tissue, and thereby provide more valuable diagnostic information. Elasticity images have been shown to be able to provide new opportunities for the detection and diagnosis of cancers in the breast [7], [16], [17], prostate [18], and liver [9], [19] and in other clinical applications [20], [21] associated with assessments of the elastic properties of soft tissue.

Ultrasound elastography, which was originally proposed by Ophir et al. in 1991 [3], has been evolving into a useful and promising technique due to its real-time capability and ease of implementation. Among the various elastographic techniques [22]–[24], quasi-static ultrasound elastography is particularly popular, whose basic steps are as follows: (1) a set of radio-frequency (RF) signals is collected from the specimen in its undeformed state; (2) the specimen is compressed by external loading, which can be assumed to be quasi-static, and another set of RF signals is recorded; (3) motion -tracking techniques, such as widely used cross-correlation techniques, are applied to estimate the displacement field between the two sets of RF signals recorded in the previous two steps; and (4) so called elastograms are reconstructed/computed from the displacement field. The term elastogram is generally used when referring to all kinds of images that display mechanical attributes of the tissue, such as the axial or lateral strains, the elastic modulus or Poisson's ratio [25].

Elastography can be broadly classified into two groups based on the mechanisms underlying the generation of the elastograms. In the first group, the relative mechanical attributes are calculated directly from experimental observations (usually tissue displacements), and axial, lateral, and/or shear strains are calculated from the tissue displacement field and then inverted to produce elastograms [26], [27]. Although this interpretation of the strain images may be affected by certain artifacts such as target hardening and bidirectional shadows [3], [28], these strain images have received considerable attention over the past 20 years because it is feasible to obtain them in real time using an ultrasound system. There is an increasing number of commercial ultrasound machines that offer elastography capabilities, which makes them capable of generating strain images in real time. In the second group, intrinsic elastic parameters are reconstructed quantitatively from the measurement data (also tissue displacements) [29]–[32]. The reconstruction process involves finding an optimal solution to an inverse problem with constraints, such as the assumption of plane-strain situation, knowledge of the boundary conditions and several other assumptions. Modulus elastograms, such as the distribution of Young's modulus, can be thus obtained with the optimal solution. Modulus elastograms can greatly suppress the artifacts in strain images, but their quality is highly dependent on the precision of displacement measurements, which can be easily destroyed by many uncertainties owing to the ill-posedness nature of the inverse problem. For example, some proposed reconstruction algorithms [22]–[24], [30] require knowledge of lateral displacements, while the robustness of many reconstruction methods could be easily affected by displacement measurements with a poor signal-to-noise ratio (SNR) [24], [29], [33].

For both the calculation of strain images and the reconstruction of elastic parameters, accurate estimation of tissue displacements is the first important step that will critically affect the image quality. This has prompted the development of different motion-tracking techniques for recovering tissue displacements during the past 2 decades [3]–[5], [9], [27], [34]. The estimation of tissue displacements is inherently a three-dimensional problem, which means that the displacement vector components physically involve all three directions (*x*-, *y*-, and *z*- axes) simultaneously and continuously. However, early methods only focus ed on axial displacement estimation because traditional ultrasound probes provide high spatial resolution along the axial direction, but poor spatial resolution along the lateral direction. A widely used displacement estimation technique is time delay estimation (TDE) [3], [27]. TDE method generally involve finding the best-matching segment in the delayed RF signal for a specific segment in the reference RF signal by computing the maximum or minimum of a pattern-matching function. Cross-correlation is the most commonly used pattern-matching function in TDE method, but several other matching techniques have also been employed, such as those based on correlation coefficients [35], hybrid-sign correlations [36], the sum of absolute differences (SAD) [37], and the sum of squared differences (SSD) [38]. The performances of these matching techniques have been comprehensively surveyed in [39], [40]. TDE provides accurate estimation of axial displacement, but it is normally time-consuming. An alternative way is to use phase-shift estimation (PSE) originating from Doppler techniques, which has the advantage of efficient calculation of displacements [5], [41], [42] and then is more feasible to implement in commercial ultrasound systems.

However, axial displacements estimated from single ultrasound RF lines are insufficient for reconstruct ing modulus elastograms [1], [30]. Previous approaches for estimat ing both the axial and lateral displacements have relied on the speckle-tracking technique [10], [43]–[46]. The main challenge in estimat ing lateral displacement s in ultrasound elastography is due to the spatial resolution being much worse along the lateral direction than along the axial direction for most ultrasound probes. Different beam-forming schemes have been proposed for improving the lateral resolution [47], [48], such as using a large beam steering angle; however, this method can only be implemented using a phased array. Different post processing techniques have also been applied to improve the quality of lateral displacement measurements, such as iterative interpolation along the lateral direction [26] or local affine transformation [49]. Another approach has been to calculate a two-dimensional (2D) displacement field in real time using the analytic minimization (AM) of cost functions that incorporate both the similarity of the amplitudes of RF signals and the displacement continuity [9]. However, lateral displacement estimation is still typically an order of magnitude less accurate than axial displacement estimation. This limitation has manifested in clinical applications, such as being unable to identify ablation lesions in patients in experiments based on lateral-strain data [9]. Researchers have tried to use the biomechanical constraint to recover lateral displacements at a high resolution from axial data. With the assumption of a constant Poisson's ratio (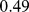), i.e. based on the biomechanical constraint of tissue incompressibility [50], lateral displacements were recovered from axial-strain measurements using the least-square technique. However, the least-square technique in this tissue -incompressibility-assumption method (TIAM) cannot perfectly eliminate measurement noise. In addition, errors will be introduced when the tissue incompressibility assumption is invalid. It is therefore necessary to develop a robust framework with a more meaningful biomechanical constraint to recover the full displacement field from the ultrasound measurements.

In this paper we present a biomechanical-model-based filtering framework for improving the quality of the full displacement field obtained from noisy and sparse ultrasound data. A modified PSE method that we proposed previous ly [42] is first used to compute the axial displacement from RF signals, and then an 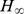 filtering algorithm [51] is applied to generate statistically optimal estimates via the assimilation of measurements with a meaningful biomechanical model constraint. After recursive filtering procedures, strain elastograms (axial-, lateral-, and shear- strain images) can be calculated from the recovered full displacement field. Synthetic data were generated to evaluate the robustness and accuracy of our strategy. Moreover, the promising results obtained from real ultrasound phantom data and clinical data show the great potential of our approach in elastography. The remainder of the paper is organized as follows: The first section gives an introduction to our framework, including the displacement calculation algorithm based on PSE, the biomechanical model and its state-space representation, and the 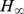 filtering procedure used for integrating displacement measurements and model prediction. The second section describes the freehand elastography experiments on synthetic data, and presents the phantom and clinical data used to evaluate the performance of our framework. The third section discusses the experimental results and the fourth section draws the conclusion s from this study.

## Methods

**Figure 1:**
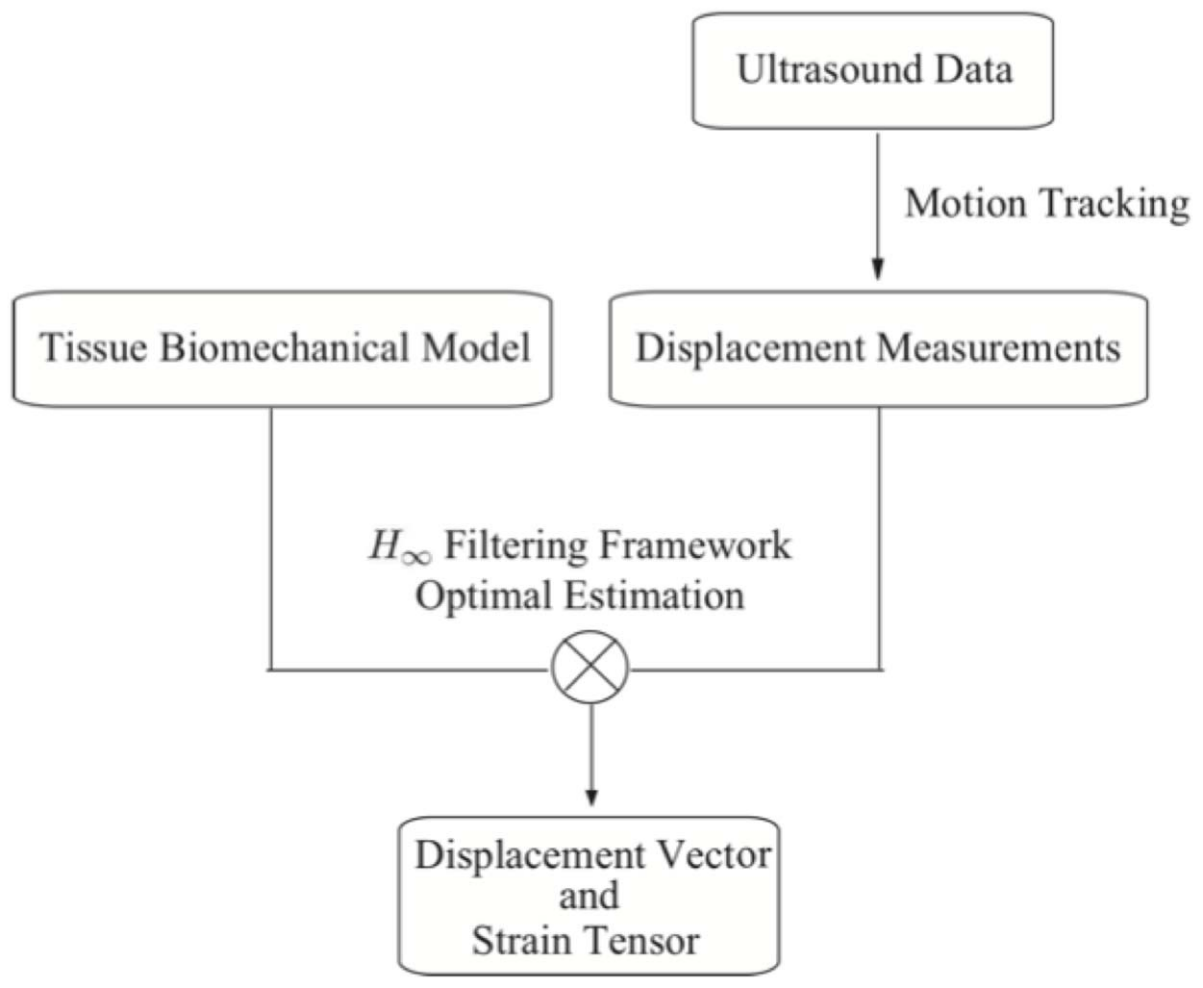
Flow chart of the algorithm.

The whole framework includes two parts, motion tracking and motion recovery, as shown in Figure 1. Axial displacements are first computed from ultrasound RF signals using the PSE motion-tracking method proposed previously by our group [42], and then axial displacements are used as observations and input to the subsequent 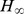
filtering strategy to reconstruct a 2D displacement field. This section briefly describes our PSE method for measuring the axial displacement, and then describes in detail the integration of the biomechanical model and the 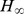 filtering strategy.

### Recovery of Displacement

We previously developed a PSE method to calculate the axial displacement of soft tissue under quasi-static compression. The ultrasound RF signals before and after deformation can be modeled in complex expressions:
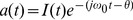
(1)

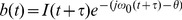
(2)where 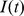 is the envelope of the RF signal, 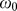 is the center angular frequency of the transducer, 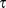 is the time lag due to deformation, and 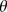 is the initial phase. The complex RF signals can be obtained by in-phase/quadrature demodulation or using the Hilbert transform.

The complex cross-correlation function of 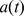 and 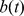 is then computed to estimate the axial displacement:
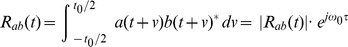
(3)where 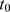 is the correlation window and superscripted asterisk denotes the complex conjugate. This expression corresponds to the output of the autocorrelation operator of a conventional Doppler system. If axial displacement 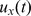 is smaller than a quarter of wavelength 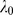, it can be obtained from phase shift 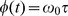 which is equal to the phase of 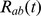:
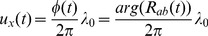
(4)where arg(*) denotes the phase of one complex number.

**Figure 2:**
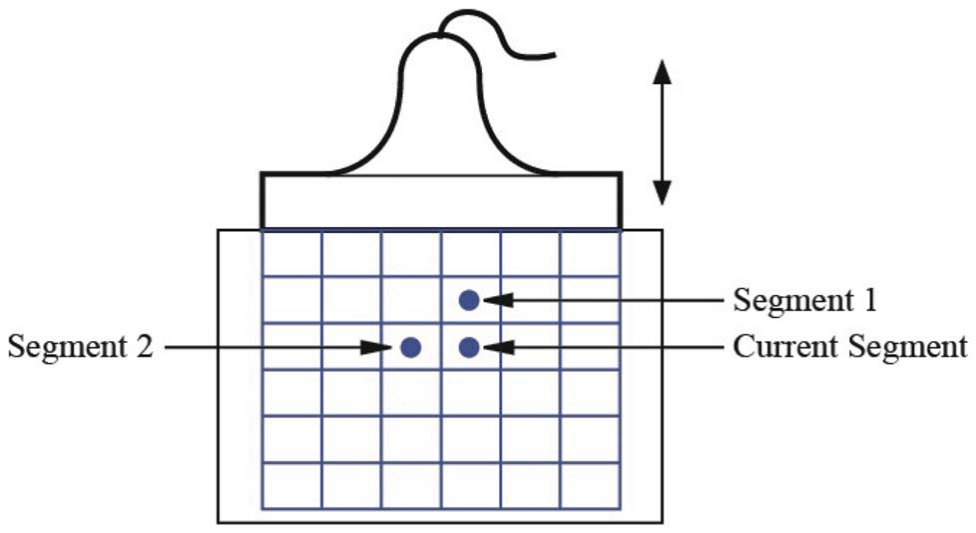
Choice of prior information.



However, phase aliasing occurs when 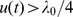, in which case the displacement cannot be calculated from Equation (4) without ambiguity. We introduced *a prior i* knowledge of the displacement (time lag 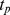) to perform phase unwrapping [42] in order to avoid the estimation error. As shown in Figure 2, the ultrasound image is divided into an appropriate number of overlapping segments. The displacements are assumed to be continuous across neighboring segment, and hence the information available from two neighboring segments can be used to predict time lag for the current segment: 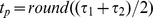. The displacement is defined as the relative movement of the segments with respect to the probe, and so the value of 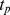 for all of the first segments in each RF line is set as zero. By introducing prior estimated time lag 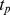, Equation (3) is rewritten as

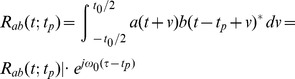
(5)Therefore, axial displacement 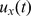 can be calculated from the following equation without ambiguity:
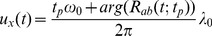
(6)


### Biomechanical Model

In ultrasound elastography, soft tissue is usually modeled as an isotropic linear elastic medium, where the elastic properties are identical in all directions and the strain/stress tensors are symmetric owing to only small deformation s occurring in response to an external force. In this case Hooke's law defines the relationship between strain and stress tensors, and it can be described by the following equations using standard tensor notation [52]:
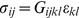
(7)where 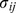 is the Cauchy stress tensor, 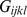 is the stiffness tensor, and 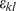 is the strain tensor. In this study we only consider ed 2D mechanical model in the Cartesian coordinate system, which makes it more convenient to describe Hooke's law in matrix form as follows:
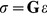
(8)where 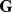 is the stiffness matrix. The displacement components along the 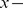 axis (axial direction) and 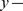 axis (lateral direction) are 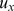 and 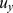 respectively, and so strain tensor 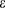 and stiffness matrix 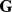 under a plane -strain condition are given by



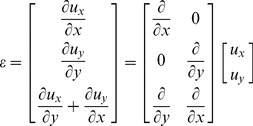
(9)

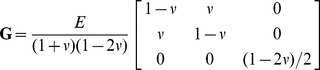
(10)where 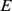 is Young's modulus and 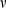 is Poisson's ratio. Equation (10) indicates that stiffness matrix 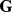 is determined by only two scalars in this work: Young's modulus (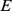) and Poisson's ratio(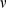).

After the constitutive law of linear elasticity is defined, the governing equation of motion expressed using components in a rectangular Cartesian coordinate system can be easily defined as follows [52]:
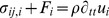
(11)where the 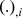 subscript is shorthand for 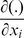, 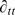 indicates 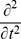, 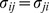 is the Cauchy stress tensor, 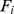 are the body forces, 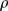 is the mass density, and 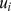 (i.e., 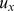 and 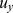) refers to the axial and lateral displacement s.

In order to use the equation of motion (Equation (11)) in this work, the finite element method (FEM) is adopted to discretize the region of interests into small elements, using a Delaunay triangular finite-element mesh. Then the nodal-displacement-based governing dynamic equation of each element is established under the minimum-potential-energy principle [52], with the obtained equations assembled into the following matrix form:
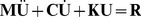
(12)where 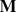, 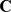 and 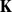 are the mass, damping and system stiffness matrices, 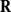 is the load vector, and 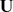 is the displacement vector. 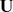 consisting of 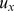 and 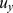 values (i.e., axial and lateral displacement s). The 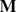, 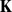 and 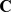 matrices were calculated in all of the experiments performed in this study using the same well-known standard FEM procedure [52]. Because the tissue density can be generally considered to be uniform over the region of interest, 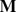 is a known function of the material density and is both temporally and spatially constant. 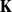 is a function of the constitutive material property, which was linear elasticity, and is related to the material-specific Young's modulus and Poisson's ratio, which were considered as temporally constants in this study. However, in our framework these two local material parameters are initialized uniformly by known knowledge, such as previously reported material properties of healthy background-tissue. Damping matrix 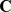 is frequency dependent, and we assume d the presence of small proportional Rayleigh damping, and so 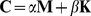 in our implementation, where 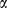 and 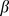 are the the mass- and stiffness-proportional Rayleigh damping weighting coefficients, respectively [52]. In practice it is difficult to determine the damping parameters because they are frequency dependent. Our assumption of Raleigh damping was based on the very low damping exhibited by biological tissues during quasi-static elastography, and fix ed the two weighting coefficients at 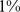.

One initialization issue of Equation (12) is how to measure the external loading vector (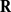) during freehand elastography. Considering the object system dynamics embodied in Equation (12), if any knowledge of the displacement vector (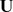) is available, it can be used as essential boundary conditions to recover the motion parameters of all other nodes. The following experiments invovling synthetic and real imaging data provided a set of displacements at nodal points of the boundary (e.g., axial displacements), and they are employed in the following fashion. Let 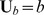 be known from the imaging data at selected sampling nodes of the boundary, then the additional constraining equation 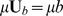 is enforced on the system dynamics through
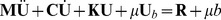
(13)where weighting coefficient 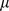 depends on the confidence of each displacement, with large 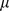 values (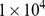 in this study) indicating highly trustworthy data points and small 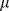 values for others. In this way it remains possible to describe the boundary condition without measuring the external force during freehand elastography. More details of this enforcement of boundary condition can be found elsewhere [52].

### Stochastic Space Representation

In previous studies of biomechanics and ultrasound elastography, the deterministic FEM has provided an efficient representation of complex tissue geometry and a convenient and effective computational framework [53], [54]. However, this method is not able to consider situations where kinematic observations should be characterized as stochastic processes. Since the imaging and imaging-derived observations are usually corrupted by various types of noise, especially for pathological situations in ultrasound elastography, it is necessary to adopt a strategy that can account for the main sources of uncertainty in the analysis of ultrasound elastography.

The stochastic FEM (SFEM) has been used for structural dynamics analysis in probabilistic frameworks [55], [56]. In the SFEM, structural material properties are described by random fields, possibly with known *a priori* statistics, and the observations and loads are corrupted by noise. In this way the stochastic differential or difference equations are combined with the FEM to study dynamic structures for which there is uncertainty in their structural parameters and/or measurements. In the analysis of ultrasound elastography, this framework based on Ito calculus from a Bayesian point of view [57] can be adopted to give optimal estimates of the kinematics state for a particular *a priori* mechanical model and *a posteriori* ultrasound imaging pairs.

In our 2D implementation, a Delaunay triangulated finite element mesh is constructed at interested area segmented manually in the first frame of ultrasound elastography. An isoparametric formulation defined in a natural coordinate system is used, where the basis functions for a trinodal linear element are linear functions of the nodal coordinates [52]. In order to apply filtering strategies to estimate both the axial and lateral displacements, we assume that the material parameters (Young's modulus 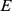 and Poisson's ratio 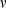)are temporally and spatially constants, which are distributed uniformly throughout the region of interest, and so Equation (12) can be transformed into a state-space representation of a continuous-time system [58]:
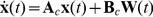
(14)where



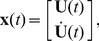
(15)

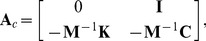
(16)

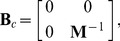
(17)

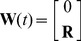
(18)


From Equation (16) to (18), 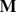, 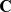 and 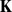 can be assembled using the standard FEM procedure [52]. The displacement was a function of time in this study, so 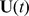 in Equation (15) represents the displacement over time.

With sampling interval 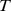, adding process noise 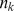 (which is not necessarily white noise), yielding the following discrete-time system equation:
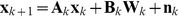
(19)where 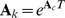, and 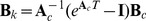. The associated measurement equation of the discrete-time system can be expressed as:
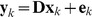
(20)where 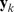 is the measurement vector, 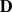 is the measurement matrix [58], and 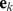 is the measurement noise. Measurement vector 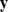 contains the sparse measurements of displacements, while state vector 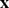 contains the full displacement components. Therefore, 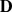 is actually a single mapping matrix that describes the correspondence between the available measurement and full displacement components. The approach based on this filtering algorithm has no specified requirements for the type of measurement. Equations (19) and (20) are thus the discrete-time state-space representation of the object dynamics of ultrasound elastography with biomechanical modeling constraints.

### 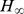 Filtering Strategy

The popular Kalman filter calculates the estimation error using the 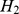 norm and minimizes the mean-square error to find the optimal estimates, while the 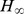 filter evaluates the error in terms of the 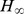 norm via the energy gain [59]:
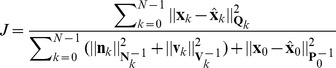
(21)where 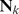, 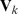, 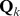, and 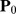 are the weighting matrices for the process noise, measurement noise, estimation error, and initial uncertainty respectively, and 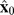 is *a priori* estimates of 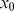. The 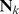, 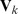, 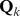 and 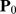 matrices that are used in the weighted norms in 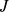 are chosen manually so as to satisfy desired trade-offs. For example, if it is known that the process noise will be lower than the measurement noise, 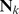 should be made smaller than 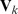 so as to de-emphasize the importance of the process noise relative to the measurement noise. Similarly, if we are more concerned about the estimation accuracy for specific elements of the state vector, or if the elements of the state vector are scaled so that they differ by an order of magnitude or more, then 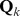 should be defined accordingly. In most related work, including that performed for this paper, 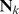, 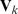, and 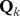 are assumed to be time-invariant constant matrices [51], [59]–[61]; therefore, in the following context, 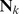, 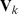 and 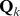 are replaced by 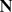, 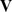 and 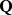, respectively. Since all the elements in the state vector (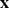) are weighted equally in this stduy, 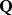 is set to be the identity matrix. Further, since 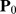 presents the initial guess of error covariance matrix, we always set one large constant (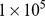) along the diagonal elements of 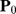 and zero elsewhere in this study. According to our experience in the following experiments, the error covariance matrix (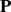) will converge after the iterations.

The denominator of 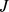 can be regarded as the energy of unknown disturbances, while the numerator is the energy of the estimation error. Considering a system given by Equations (19) and (20), the 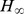 filtering process involves searching the optimal estimates of 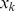 that satisfy the following performance measure:
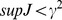
(22)where 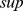 means the supremum and 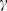 is a positive constant that represents a prescribed level of noise. The robustness of the 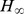 estimator is based on it yielding an energy gain of less than 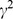 for all bounded energy disturbances irrespective of where they are. We found that the performance of our filtering strategy is acceptable when 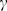 = 1 in all the following experiments.

Many strategies can be used to implement the 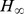 filter [51], [59]. Here, we adopted a game -theory algorithm that does not require the checking of the positive definiteness and inertia of Riccati difference equations (Equation (23)) for every step [62]. In our implementation strategy the 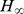 filtering algorithm for the system described by Equations (19) and (20) with the performance criterion in Equation (22) consists of the following procedures:

1. Compute the FEM matrices; that is 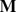, 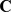, and 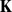 in Equation (12).
2. Initialize the 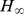 filtering algorithm by choosing appropriate 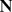, 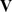, 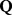, and 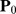

3. Start the following iterative procedure:

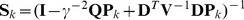
(23)

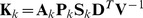
(24)
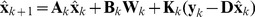
(25)

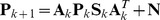
(26)where 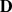 is the measurement matrix in Equation (20), and 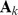 and 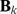 are the matrices in Equation (19).

It is obvious that the above 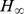 process has a Kalman-like structure. However, the 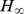 filter ing strategy does not require any prior knowledge of noise statistics, and it would be more appropriate for practical problems.

### Experiments

This section uses three groups of data–synthetic data, phantom data, and clinical data–to evaluate the performance of our filtering strategy. First, a finite -element model was constructed in ABAQUS (3DS Simulia Corporation, Waltham, USA) with a hard circular inclusion embedded in a soft background to generate the synthetic data: the displacement map was obtained by applying axial loading to the sample surface as the ground truth, and various levels and types of white noises were added to the simulated axial and lateral displacements in order to construct measurements for the 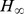 filtering strategy. The availability of the ground truth in experiments using synthetic data makes quantitative evaluation s of the performance of our filtering strategy easy, such as the calculation of the SNR. Moreover, for elastography using a one -dimensional probe, because the quality of the reconstructed lateral displacement can reflect the capabilities of our proposed strategy, our experiments using synthetic data focused on examining the quality of estimated lateral displacement through the 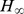 filtering strategy. Then, a PC-based ultrasound system with open access to the RF signals (Ultrasonix RP, Ultrasonix Medical Corporation, Burnaby, BC, Canada) was employed to collect experimental data from an elasticity QA phantom (model 049, CIRS Technology, Norfolk, VA, USA). For this set of data, our PSE method was used to estimate tissue internal displacements from the ultrasound RF data [42], with only the axial component of displacement field used as a measurement in our model-based filtering framework. Although the measurement s in this experiment only constitutes information for the axial direction, our proposed filtering strategy can still recover a high-quality displacement field and strain elastograms in both the axial and lateral directions. We also conducted experiments using data available online [9] from Johns Hopkins University (JHU). These data contain both axial and lateral displacement components estimated from the ultrasound RF data using the 2D AM method. Our model-based framework was then used to estimate the displacement field at a high spatial resolution by using both the noisy axial and lateral displacement components generated by the 2D AM method as measurements. The measurement s used in this experiment contain ed more information than the one in the earlier experiment, which meant that our filtering strategy could generate better results. The corresponding strain elastograms were also compared with those directly calculated from displacement measurements [9].

### Synthetic Data

**Figure 3:**
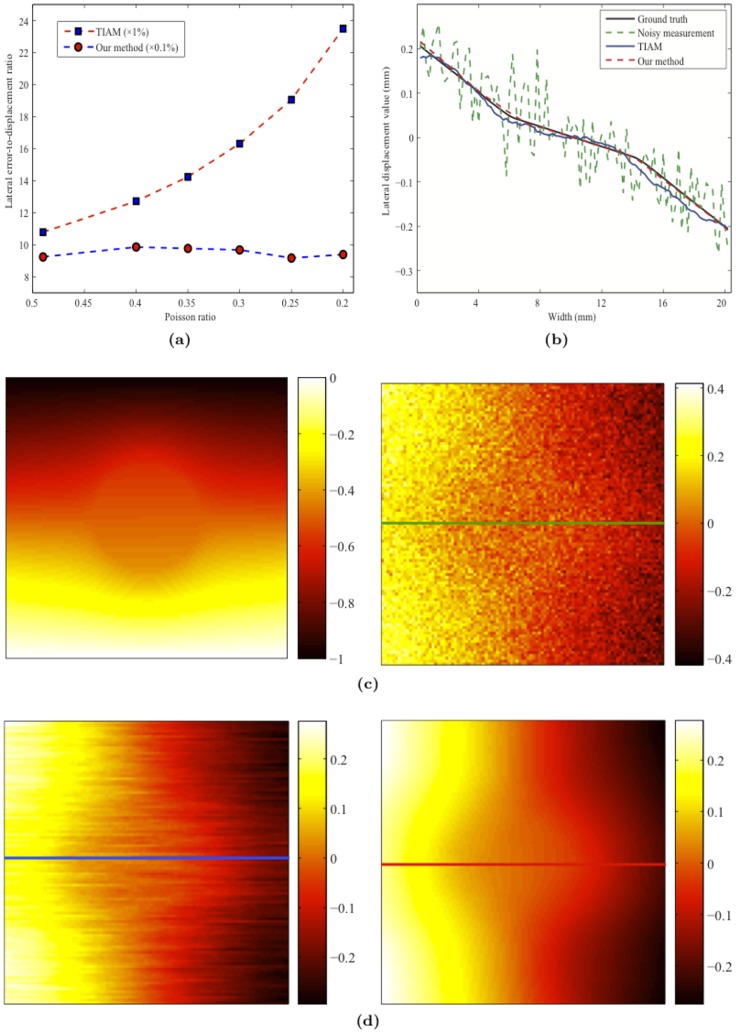
Performance comparison: (a) 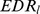 versus Poisson's ratio for the two methods. (b) Lateral displacement profiles at the same depth that is marked by the colorful lines in (c) and (d); (c) Measurements: axial displacement with low noise (left) and lateral displacement with high noise (right); (d) Results for TIAM (left) and our method (right).

**Figure 4:**
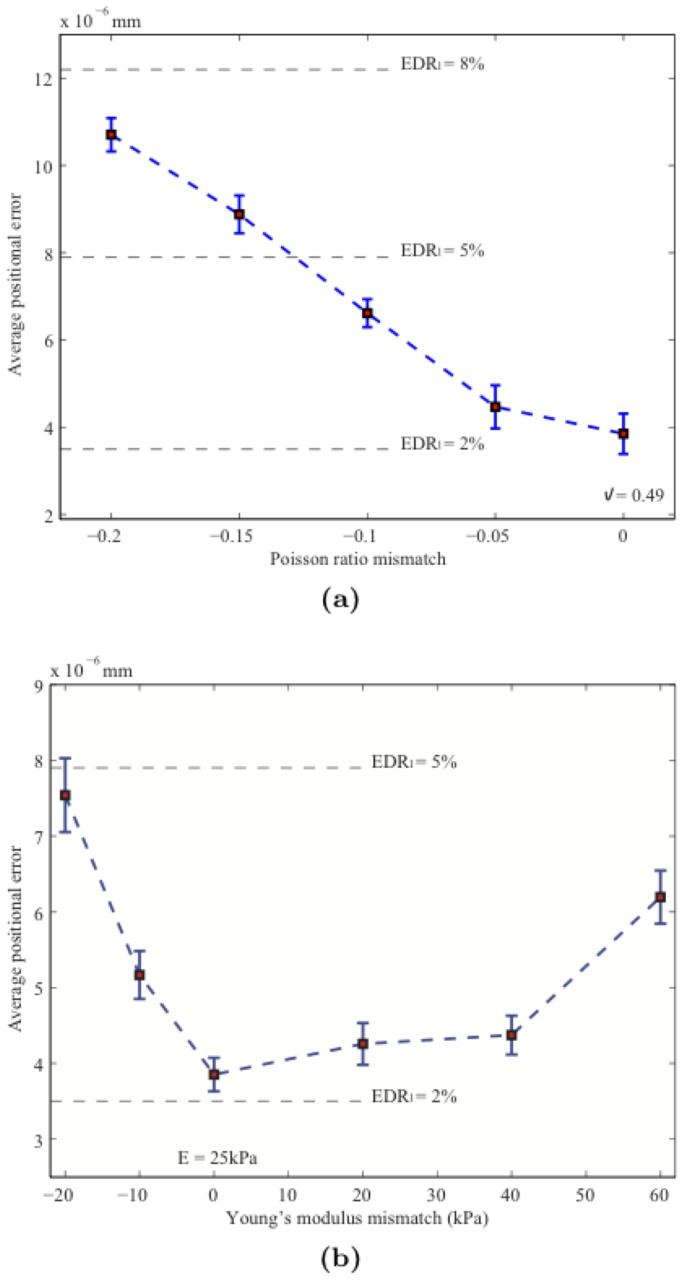
Effects of Poisson's ratio mismatch (a) and Young's modulus mismatch (b).

A 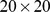 mm rectangular object containing a circular inclusion with different material properties (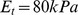 and 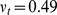 for the circular inclusion, and 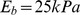 and 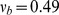 for the background), was constructed in ABAQUS. The following boundary condition was applied: the object was loaded by uniaxial compression so that it moved 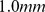 (i.e., the deformation ratio was 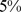) downward with only its bottom fixed. The corresponding displacements calculated by ABAQUS were used as the ground truth. Different types and levels of white noise were added to the simulated displacements as the measurements for our model-based filtering framework. Two groups of simulations were implemented for generat ing the synthetic data:

1. Using the same boundary condition as described above, six sets of displacements were obtained for Poisson's ratio of 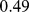, 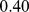, 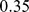, 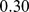, 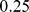, and 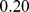 (for both the inclusion and the background). Since the spatial resolution of the RF data is higher in the axial direction than in lateral direction, the quality of the axial -displacement measurements should always be higher in quasi-static ultrasound elastography. Therefore, it is reasonable to add high noise levels to the synthetic lateral displacements; SNR values of 30 and 10 dB were used for the axial and lateral displacement component s, respectively. This experiment used both axial – and lateral -displacement measurements in our method. The process noise (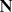) was one diagonal matrix (diag(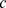, 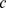,..., 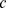), 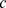 = 1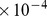), and 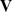 was another one diagonal matrix (diag(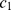, 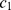,..., 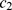, 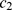), 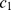 = 1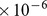 for axial displacement measurements, 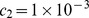 for lateral displacement measurements) in this experiment. Both the TIAM and our model-based filtering method were applied to recover the lateral displacements from the simulated measurements. Noted that this paper focus es on the estimation of strain from quasi-static ultrasound elastography; the estimation of lateral displacement is always very difficult due to the poor resolution of the ultrasound probe in that direction. Therefore, the ability to recover high-quality data for the lateral displacement field is the key indicator of the performance of strain -estimation algorithms used for quasi-static ultrasound elastography. The ability of our proposed method to recover the lateral displacement is quantified using the lateral error-to-displacement ratio (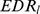):
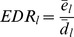
(27)where 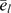 is the mean of the absolute values of nodal lateral-displacement error, 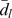 is the the mean of the absolute values of nodal lateral-displacement value. The ability to recover the lateral displacement recovery of two algorithms (the TIAM and our model-based filtering method) are compared in this experiment. The 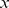-axis is the Poisson's ratio and 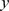-axis is the lateral error-to-displacement ratio in Figure 3(a). Figure 3(a) provides the 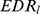 curves for the two algorithms, and the true magnitude of the 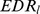 in our method should be equal to the value on the curve multiplied 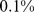. Furthermore, the 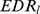 curve for the TIAM goes up when Poisson's ratio descreaes, whereas the 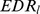 curve for our method does not change much. Figure 3(b) shows lateral displacement profiles at the same depth indicated by the colored lines in the following Figure 3(c) and (d). As shown in Figure 3(b), the lateral -strain profile generated by our method is much closer to the ground truth. One set of simulated measurements (for a Poisson's ratio of 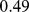) is shown in Figure 3(c). The left panel in Figure 3(c) displays the synthetic measurement s of the axial displacement. The high spatial resolution along the axial direction of the ultrasound probe means that the quality of the synthetic measurement s should be much higher for the axial displacement than for the lateral displacement, as is evident in the right panel of Figure 3(c). The lateral displacements recovered by the TIAM and our method are shown in Figure 3(d), which clearly indicates that the quality of recovery is much higher for our method.

2. The second experiment evaluated the effect of modeling mismatch or external disturbances on the performance of our method. Unlike the earlier experiment, in this experiment only the axial displacement (with SNR = 30 dB) was used as the measurement, and the average nodal position error (i.e., the differences between the estimates and the true values calculated by ABAQUS) was calculated to evaluate the accuracy of the estimation. The process noise (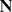) was one diagonal matrix (diag(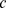, 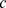,..., 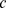), 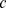 = 1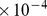), and 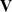 was another one diagonal matrix (diag(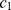,..., 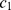), 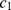 = 1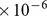 for axial displacement measurements) in this experiment. The ability to recover high-quality lateral -displacement measurements is important in elastography because of the poor lateral spatial resolution of the probe. In this experiment 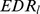 was used as one performance indicator of this ability. In order to better demonstrate this ability, we only use d the measurements from the axial direction; that is, the measurements did not contain any information from the lateral direction. In this paper the linear elasticity is adopted as the mechanical property. Therefore, the mismatch of the model involves evaluating the mismatch es of Young's modulus and Poisson's ratio, which are two key factors of the linear elasticity. In the following experiments the mismatches of Poisson's ratio and Young's modulus are evaluated, respectively. The ground truth of Poisson's ratio in Figure 4(a) is 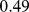. The difference in Poisson's ratio is equal to the used value minus the ground truth. The *x*-axis of Figure 4(a) is the difference in Poisson's ratio while the *y*-axis is the average nodal position error. The 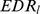 line is also drawn in Figure 4(a) to indicate the quality of the estimated lateral displacement. Figure 4(a) shows that the nodal position error increases with the mismatch of Poisson's ratio, but 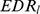 is below 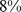 when the mismatch is 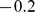. In the experiment of the mismatch of Young's modulus, for the ground truth Young's modulus is 80kPa for circular inclusion and 25 kPa for the background. However, in our 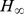 filtering strategy Young's modulus is considered as a constant parameter both spatially and temporally since we assume that we do not know the location of the inclusion during the filtering process. Young's modulus is therefore initialized at the same value over the whole area in our filtering strategy, and the value of Young's modulus is initialized to 25 kPa. The difference of Young's modulus between the ground truth and used value in Figure 4 is equal to that used value minus the background value. The *x*-axis of Figure 4(b) is the difference of Young's modulus and *y*-axis is the average nodal position error. Figure 4(b) shows that when the mismatch of Young's modulus is –20, the average nodal position error increases rapidly, while when the mismatch of Young's modulus goes to 60, the average nodal position error increases slowly. However, 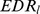 is still within the acceptable zone, as indicated in Figure 4(b). In addition, different types and levels of external noise were added to the simulated axial displacements to generate different types of measurements. After the estimated nodal positions were obtained, the mean and standard deviation values for the nodal position errors were calculated; the results are complied in Table 1. In this test of different types of external noises, our robust model-based framework can produce rather similar results from measurements with various types of additive noises (mean values for the nodal position errors do not change largely at the same level of external noise, as shown in Table 1), but for different levels of external noise, the estimated results still changes (mean values for the nodal position errors change across different levels of external noise, as shown in Table 1).

**Table 1:** Comparison of average nodal position errors (mean 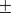 standard deviation values) derived from the 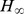 filtering results for various types of measurement noise.

	Average Positional Error (×10^−6^)	
Noise Type	SNR 20 dB	SNR 15 dB
Gaussian	3.869±0.075	7.369±0.365
Uniform	3.440±0.076	6.963±0.439
Poisson	3.586±0.187	7.089±0.326
Rayleigh	3.854±0.086	7.070±0.219
Exponential	3.473±0.286	7.284±0.734

**Figure 5:**
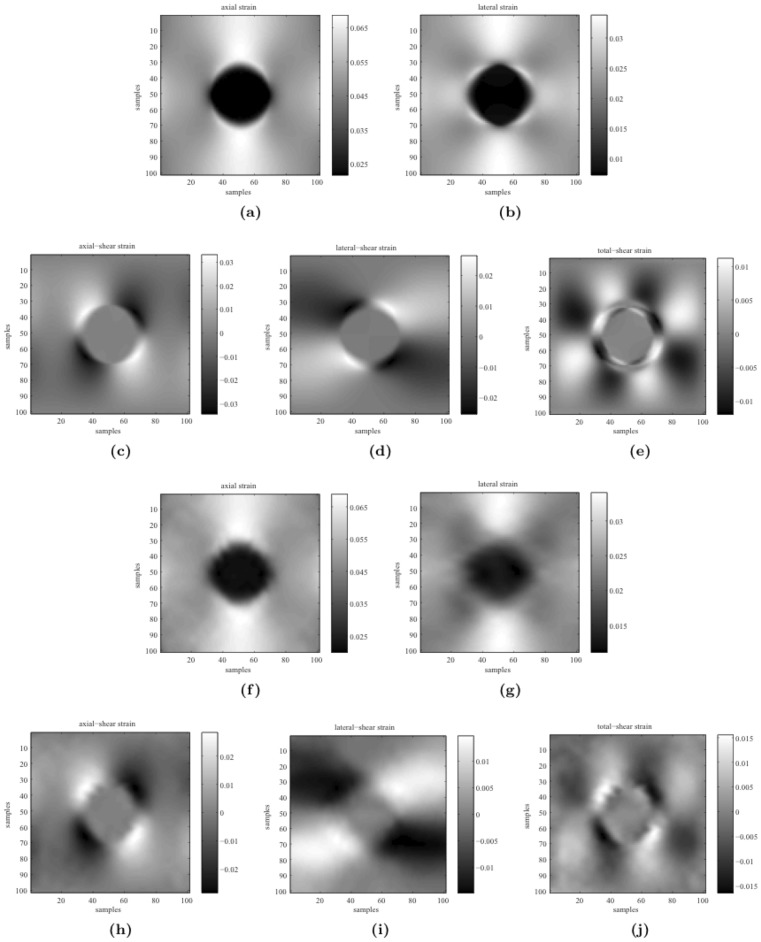
Ground-truth data: (a) axial strain, (b) lateral strain, (c) axial-shear strain, (d) lateral-shear strain, and (e) shear strain. Reconstructed strain tensors: (f) axial strain, (g) lateral strain, (h) axial-shear strain, (i) lateral-shear strain, and (j) shear strain.

Based on the recovered axial and lateral displacements, all components of the strain tensor can be reconstructed, as shown in Figure 5. These results demonstrate that our model-based framework can accomplish a high-quality tissue motion recovery (i.e., axial and lateral displacements) from the sparse and noisy measurements. However, in order to reduce the computational consumptions, we only choose 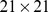 nodal displacements as the measurements for our method in the simulations.

### Phantom Data

**Figure 6:**
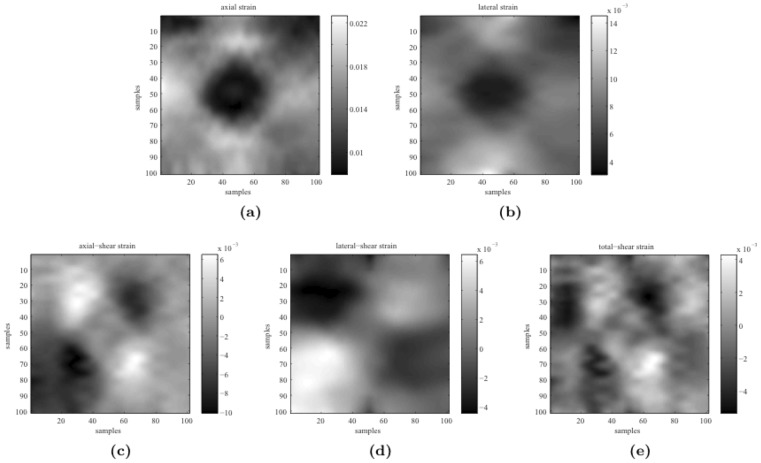
Components of the strain tensor calculated from the recovered displacements: (a) axial strain, (b) lateral strain, (c) axial-shear strain, (d) lateral-shear strain, and (e) shear strain.

Two groups of phantom data are used to verify the performance of our model-based filtering approach. In the first group, RF data were obtained at a sampling rate of 20 MHz from the Ultrasonix RP system (Ultrasonix Medical Corporation) comprising a transducer having a center frequency of 5 MHz. All of the ultrasound data were collected using freehand elastography on an Elasticity QA Phantom (CIRS Technology). The axial displacements were first obtained using our PSE method from the RF signals as measurements to our method, and the full displacement field (both axial and lateral displacements) was then recovered at a high spatial resolution from these axial-displacement measurements through our model-based filtering framework. The process noise (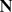) was one diagonal matrix (diag(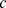, 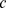,..., 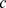), 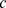 = 1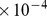), and 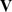 was another one diagonal matrix (diag(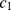, 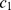,..., 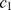), 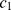 = 1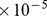 for axial displacement measurements) for the first group of phantom data. The following strain tensors were calculated based on the recovered axial and lateral displacements. Figure 6 shows that the obtained lateral-strain image s were clear, and that the shear-strain images could still be identified. These high-quality strain images were reconstructed using our filtering algorithm with only measurement of the axial displacement.

**Figure 7:**
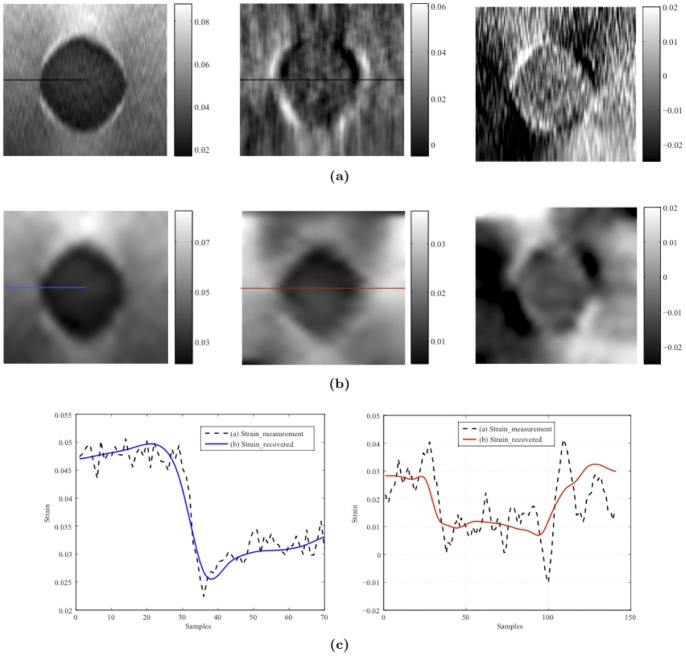
Comparison between our method and other method: (a) Axial-, lateral-, and shear-strain images (left to right) calculated from displacement measurements directly. (b) Axial-, lateral-, and shear -strain images (left to right) calculated from displacements estimated by our filtering strategy. (c) Comparison of the axial-strain (left) and lateral-strain (right) profiles of images from two groups.

The second group of data, both the axial and lateral displacements estimated by the 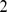
D AM method, were provided [9] by JHU. However, the lateral displacements and strain maps are very noisy, as shown in Figure 7(a). In this experiment we directly use d the axial and lateral displacements from the 2D AM method as the measurement input to our method. The process noise (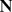) was one diagonal matrix (diag(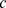, 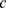,..., 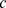), 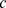 = 1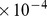), and 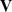 was another one diagonal matrix (diag(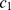, 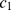,..., 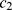, 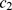), 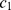 = 1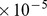 for axial displacement measurements, 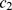 = 1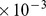 for lateral displacement measurements) for the second group of phantom data. Applying our model-based method to the noisy displacements data from JHU recovered a high-quality displacement field and strain tensors. Figure 7(b) shows that the axial-, lateral-, and shear-strain maps as calculated by our model-based method were of higher quality than the JHU results. Figure 7(c) compares the strain profiles obtained at the same locations of our images and the JHU images, which shows that the strain profile generated by our model-based method is smoother than that of the JHU method. The contrast to noise ratio (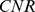) [9] was used to quantitatively compare the qualit ies of the strain images generated by our filtering strategy and the previously developed 2D AM method [9]. As shown in Figure 7, the 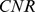 of the axial- and lateral-strain images estimated by the 2D AM method were 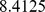 and 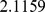, respectively; the corresponding values from our model-based method were 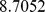 and 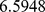.

### Clinical Data

**Figure 8:**
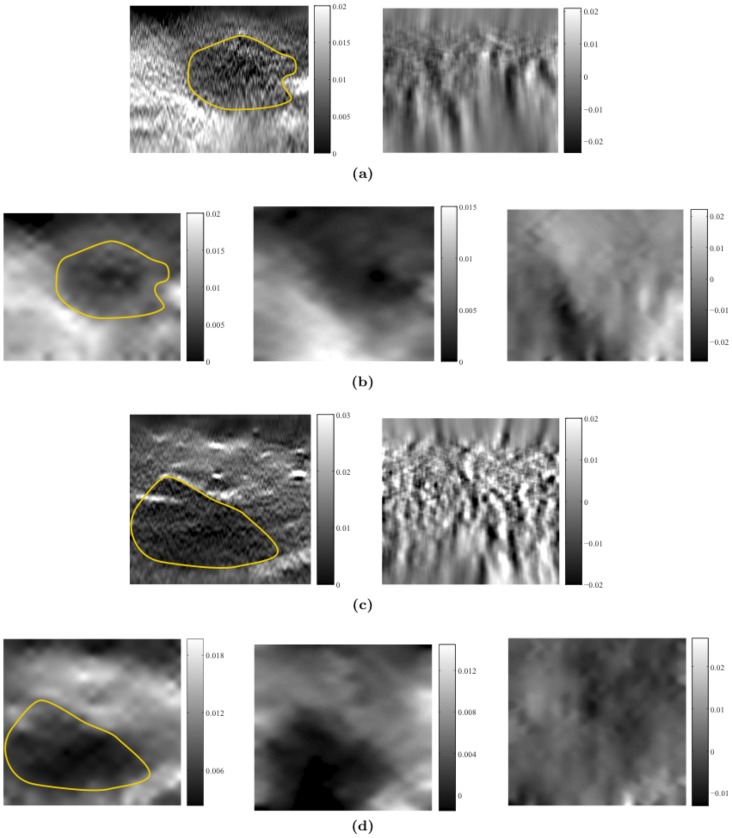
Experiment on clinical data. Data for patient 1: (a) axial- and lateral-strain images (left to right) calculated from displacement measurements directly; (b) axial-, lateral-, and shear-strain images (left to right) calculated from displacements estimated by our filtering strategy. Data for patient 2: (c) axial- and lateral-strain images (left to right) calculated from displacement measurements directly; (d) axial-, lateral-, and shear-strain images (left to right) calculated from displacements estimated by our filtering strategy. The lesions were segmented manually by the yellow lines in all four axial-strain images.

Data from two groups of patients from JHU [9] were adopted as clinical trials in this study. These data were collected from patients undergoing open surgical RF thermal ablation for liver cancer who were enrolled between February 6, 2008 and July 28, 2009. The clinical status es of these patients are described in detail [9]. The ultrasound experiment s were performed with the approval of the Health Science Research Ethics Committee of JHU, and the participants provided written informed consent before beginning the experiment [9]. The strain images corresponding to the axial and lateral displacements as reconstructed from RF data by the 2D AM method [9] are shown in Figure 8(a) and (c). The lesions were already segmented manually by yellow lines in both the axial-strain images. However, the lateral-strain elastograms generated by the JHU method did not provide valuable clinical information because the noise in their recovered lateral displacements was not compressed. Therefore, our model-based framework was applied to recover the full displacement field from the displacement measurements estimated by the 2D AM method. According to previous reports on the mechanical properties of liver tissue, Young's modulus of healthy liver tissue is around 6kPa [63]. Hence, in this work Young's modulus was set to be 6kPa and Poisson's ratio was set to be 0.49 initially. The process noise (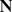) was one diagonal matrix (diag(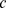, 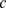,..., 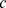), 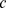 = 1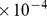), and 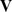 was another one diagonal matrix (diag(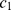, 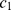,..., 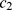, 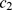), 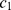 = 1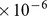 for axial displacement measurements, 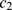 = 1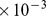 for lateral displacement measurements) in this experiment. All of the components of the strain tensor were then calculated from the recovered displacement field, as shown in Figure 8(b) and (d). The lesions were also segmented manually by yellow lines in both the axial-strain images. Our elastograms made it possible to identify the lesion from lateral-strain maps, but we could not identify any structures in the shear-strain maps that could be clearly attributed to the lesion.

## Discussion

This study used a stochastic filtering strategy to absorb the biomechanical model constraint and then perform tissue motion recovery in ultrasound elastography. The main advantage of our filtering strategy is its capability of generating a a high-resolution displacement field (in both axial and lateral directions) in 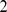
D elastography. We discuss the performance of our filtering strategy with the biomechanical constraint in term of robustness, biomechanical constraint, initialization, computational cost, and the limitations in the following context.

### Robustness of 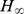 Filtering Strategy

The kernel of our proposed approach is the 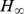 filtering strategy, which can better deal with internal and external disturbances/noise in the elastography; our 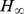 approach only requires *a priori* error bounds of disturbances/noises for the iterative recovery process, whereas most previous model-based approaches have require d more information about the disturbances/noise, such as the statistical properties [24]. Compared with the popular Kalman filtering paradigm [51], the 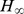 filtering strategy makes no assumptions about external noise statistics, which makes it more appropriate for certain practical problems where the disturbances/uncertainties are unknown and non-Gaussian. Furthermore, unlike the conventional filtering frameworks (e.g., median or mean filtering) that are normally employed to compress the noise included in image data, and which do not consider *a priori* information, our filtering framework recursively assimilate sparse measurements and the biomechanical-model-based constraint, and eventually generate physically meaningful optimal estimates, thereby obtaining a full and smoother displacement field. Noise is compressed in our filtering framework via the efficient determination of optimal results using the 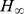 filtering algorithm under the biomechanical model constraint. In order to better understand the robustness of our proposed algorithm, we also examine the sensitivity of the property of external noise using our filtering strategy. The robustness of the proposed filtering strategy to external noise was evaluated by adding different types and levels of measurement noise to the simulated measurement. Our filtering framework was still able to recover the displacement field with a similar quality from measurements containing various types of additive noise, as indicated by the mean value s listed in Table 1. However, the standard deviations of nodal position error vary with different types of noise in Table 1, which might imply that the variation or dispersion of nodal position error from the mean value would vary with different types of noise. We could inspect the change of standard deviation of nodal position error in future work for deeply understanding the performance of the 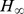 filtering algorithm in ultrasound elastography.

### Benefit of Biomechanical Constraint

The first of our experiments on synthetic data test ed the tolerance s of the TIAM and our method to variation of Poisson's ratio of the object under investigation. The TIAM gives a poor 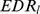 when Poisson's ratio of the object deviates from 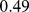 because of the assumption of tissue incompressibility. In contrast, our method exhibits high accuracy and stability, as indicated by the 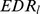 value of our method being one order of magnitude smaller than that of the TIAM (see Figure 3(a)). Our experiments indicated that a result with 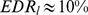 is acceptable, while one with 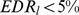 can be considered acceptablly accurate. The 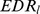 of the TIAM was around 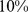 when Poisson's ratio of the object equal ed 0.49, whereas the 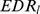 of our method was lower than 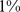, as shown in Figure 3(a).

The second experiment on synthetic data investigated the tolerance of our method to modeling mismatch and external noises. The model-data mismatch was defined as be the difference between the ground truth and the model used in our filtering strategy, such as in the boundary conditions, Young's modulus, or Poisson's ratio. Since material properties play a key role in the biomechanical modeling framework, we first examine d the effect of material-properties mismatch. Because the adopted biomechanical model is linear elasticity, the effect of the mismatch of Young's modulus and Poisson's ratio was examined in our experiments. Furthermore, since the measurements of lateral displacement provided by elastography are always sparse due to the poor spatial resolution of the ultrasound probe, the ability to recover high-quality lateral displacements was adopted as an important performance indicator in our experiments. The impact of the mismatch of Poisson's ratio on the recovery of the lateral displacement was found to be acceptable over a considerable range of modeling mismatch es (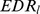 = 2
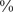
–8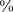; Figure 4.(a)), while the mismatch of Young's modulus did not markedly affect the quality of lateral experiments estimated by our filtering strategy (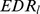 = 2
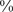
–5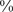). The benefit of integrating the biomechanical model into the 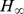 filter is that this greatly improves the quality of the obtained displacement field.

For the first group of phantom data, a 2D displacement field was recovered at a high spatial resolution only from these axial-displacement measurements through our model-based filtering framework. The strain tensors were calculated based on the recovered axial and lateral displacements. Figure 6 shows that the obtained lateral-strain images were clear, and that the shear-strain images could still be identified. Previous studies [22]–[24] found that the shear-strain images were difficult to obtain due to poor quality of the lateral displacement data. For the second group of phantom data, since both axial- and lateral-displacement measurements from the 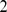
D AM method [9] are already available, it is valuable to compare the 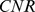 of the results obtained using both algorithms; that is, 2D AM method and our method. This phantom experiments demonstrated that our method provided better 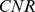 values, especially in lateral -strain image s. The strain profiles shown in Figure 7(b) also demonstrate better smoothness and higher contrast for our model-based filtering strategy. Our experiments demonstrated that the 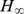 filtering strategy with the biomechanical constraint was able to recover high-quality strain images, e.g., axial- and lateral-strain images) and shear images, which have been difficult to obtain in previous studies.

The effectiveness of our filtering strategy with biomechanical strategy in estimating lateral displacements was also extensively examined in the third groups of data. The obtained images indicate that the quality of the displacement field and strain maps was greatly improved using our model-based method. Lateral-strain images, which are usually severely degraded by the noise of lateral displacements, can be recovered with higher quality and have shown some prospective clinical implications. The recovered axial and lateral displacements can be used to reconstruct all components of the strain tensor, as shown in Figures 6 and 8.

### Initialization Issues

The measurements, the number of iterations and the initialization of the external noise, Young's modulus, and Poisson's ratio are all important in our filtering strategy. The measurements represent information about the examined object, but our method does not explicitly require the type of measurements. Our filtering strategy can recover the full displacement field under the biomechanical modeling constraint. As evident from the experiments involving synthetic data (the tolerance of noise in Table 1) and phantom data (first group of data in Figure 6), our filtering strategy is still able to recover meaningful strain images in both directions. In order to acquire a strain image of high quality, we simply performed 100 iterations for the filtering procedures (i.e., Equations (23) to (26)).

The experiment on synthetic data indicates that a convenient guideline for the initialization of our filtering strategy can be easily concluded as follows:

1. Since most biological tissues are incompressible materials, Poisson's ratio for our filtering strategy can be initialized to 0.49;
2. A smaller value of Young's modulus has a larger impact on the recovery of the lateral displacement (see Figure 4(b)). Since Young's modulus of background tissue (i.e., the healthy tissue) can be easily obtained before the filtering process, this should be used as the lower limit for the initial value of Young's modulus for our filtering algorithm;
3. Although most of the parameters of the 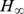 filtering strategy were fixed in this study, it is not a fully automated algorithm. Hence, the initialization discussed above should be adjusted according to each cases, weighting matrices 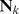, 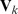, 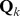, 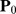, and the noise attenuation level (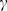) should be carefully adjusted by the designer to obtain the desired estimation performance. We could consider to adaptively adjust those parameters during the iterations of the 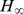 filtering strategy in future work. Further, more discussion of the adjustment of these weighting matrices can be found in Dan Simon's book and papers [59]–[61] and elsewhere [51].

### Computational Cost

The computational cost of our method is the high in current implement scheme. In the synthetic data experiments performed in this study we only used measurements on 21
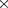
21 sample nodes. Although the spatial resolution of the recovered strain image is actually limited by the number of nodes, the quality of the estimated strain images generated by our model-based filtering strategy is not yet compromised. Furthermore, we can take advantage from developing computing power technologies, such as GPU, to accelerate our filtering strategy in future study.

### Limitations of Current Work

One limitation is that while shear-strain images may provide information about the bonding at tissue interfaces, which is clinically useful in differentiating the tumor type (benign or malignant), however, the images of shear strain did not provide any useful clinical information. This issue that should be carefully studied in the near future. Furthermore, the displacement quantification is heavily dependent on the number of elements in and quality of the finite element mesh. If the input data are reasonably accurate, increasing the number of mesh element s will definitely improve the solution accuracy; however, the current experiments indicate that this could make the computational cost unacceptable. Analysis on the computational consumption of our filtering strategy will be studied in the near future, and methods for reducing the computation cost will need to be applied before it could be implemented in a real-time ultrasound system.

Several other key issues also remain to be addressed. Since most soft tissues are nonlinear viscoelastic materials, the linear isotropic constructive equations do not properly describe their mechanical behaviors. Further research based on nonlinear material model would be useful for estimating the displacement field from ultrasound measurements. Of equal importance, we should also point out that the distribution of tissue elastic properties in the region of interest is generally not unique, and the performance of our current framework still depends on the appropriate initialization of the parameters of the biomechanical model. The chosen values must be as realistic as possible in order to acquire accurate results. As a continuation of this work, a future study will focus on an extended filtering framework that can estimate tissue displacement and elastic parameters simultaneously. Such a simultaneous-estimation framework is expected to further reduce the dependency on the choice of initial parameters. More work on the clinical application of shear-strain imaging is also expected. Further experiments should also be undertaken to more deeply evaluate the displacement values recovered by our framework. In these experiments the recovered displacements will be used to reconstruct the tissue elasticity modulus, which is likely to be more affected by the quality of the displacement field. Thus, our framework is predicted to be useful in the reconstruction of tissue elasticity.

### Conclusion

Overall, our model-based framework can accomplish a high-quality tissue motion recovery (i.e., the axial and lateral displacements) from the sparse and noisy measurements. Furthermore, our proposed filtering framework has shown potential in recover ing meaningful strain maps from available ultrasound RF data.
